# Cavotricuspid isthmus ablation using a pentaspline pulsed field ablation catheter: feasibility and acute results

**DOI:** 10.1093/europace/euae262

**Published:** 2024-10-22

**Authors:** Corentin Chaumont, Pierre Ollitrault, Arnaud Savoure, Raphael Al Hamoud, Jonaz Font, Helene Eltchaninoff, Paul Milliez, Laure Champ-Rigot, Frederic Anselme

**Affiliations:** Department of Cardiology, Rouen University Hospital, 1 Rue de Germont, 76031 Rouen, France; UNIROUEN, INSERM U1096, 22 Boulevard Gambetta, 76183 Rouen, France; Department of Cardiology, Caen University Hospital, Caen, France; Department of Cardiology, Rouen University Hospital, 1 Rue de Germont, 76031 Rouen, France; Department of Cardiology, Rouen University Hospital, 1 Rue de Germont, 76031 Rouen, France; Department of Cardiology, Caen University Hospital, Caen, France; Department of Cardiology, Rouen University Hospital, 1 Rue de Germont, 76031 Rouen, France; UNIROUEN, INSERM U1096, 22 Boulevard Gambetta, 76183 Rouen, France; Department of Cardiology, Caen University Hospital, Caen, France; Department of Cardiology, Caen University Hospital, Caen, France; Department of Cardiology, Rouen University Hospital, 1 Rue de Germont, 76031 Rouen, France; UNIROUEN, INSERM U1096, 22 Boulevard Gambetta, 76183 Rouen, France

**Keywords:** Pulsed field ablation, Atrial flutter, Cavotricuspid isthmus, Atrial fibrillation

## Introduction

The efficacy and safety of pulmonary vein isolation (PVI) using pulsed field ablation (PFA) have been largely demonstrated.^1–3^ During atrial fibrillation (AF) catheter ablation, additional cavotricuspid isthmus (CTI) ablation is often required.^4^ In patients undergoing PVI using the pentaspline PFA catheter (Boston Scientific Inc., Menlo Park, USA), it remains unclear whether CTI ablation can be achieved using the same catheter. To address this question, we prospectively included all consecutive patients undergoing combined PVI and CTI ablation with the pentaspline PFA catheter in two academic hospitals from June 2023 to March 2024. All patients provided informed consent, and the study was approved by the local institutional review board.

## Technical approach

**Figure 1:**
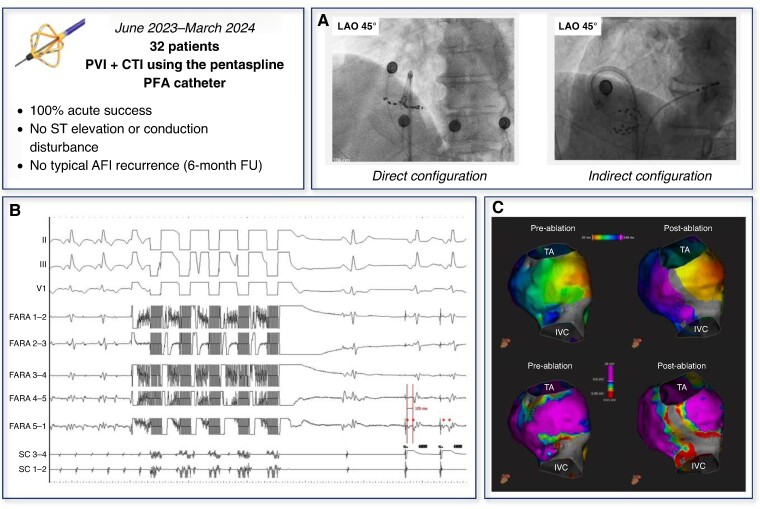
(*A*) Pentaspline PFA catheter positioning during CTI ablation. (*B*) Sinus rhythm restoration observed after the first PFA application. Note the widely separated (105 ms) local double potentials alongside the ablation line during proximal CS pacing (stars and lines). (*C*) 3D electroanatomical map of the right atrium pre- and post-CTI ablation using the pentaspline PFA catheter in indirect ‘basket’ configuration. Activation maps pre- and post-ablation (top panels). Early activation is displayed as red and late activation as purple. Note the propagation block on the CTI and the late activation on the lateral side of the ablation line during proximal CS pacing. Voltage maps pre- and post-ablation (bottom panels). Normal bipolar voltage is displayed as purple. Low voltage areas are displayed across the rest of the color scale. Note the extensive low voltage (<0.05 mV) on the CTI with normal voltage (>0.5 mV) in the lateral and septal regions of the CTI. AFl, atrial flutter; CTI, cavotricuspid isthmus; IVC, inferior vena cava; PFA, pulsed field ablation; PVI, pulmonary vein isolation; TA, tricuspid annulus.

All procedures were performed under general anaesthesia with uninterrupted oral anticoagulation. Pulmonary vein isolation was performed as previously described.^5^ The pentaspline catheter was then positioned in the right atrium, and a quadripolar catheter was positioned in the coronary sinus (CS). Cavotricuspid isthmus ablation was performed during CS ostium pacing for patients in sinus rhythm or during typical atrial flutter confirmed by entrainment manoeuvres. Based on anatomy and operators’ preference, the pentaspline catheter was positioned on the CTI using a direct approach in deployed configuration (direct ‘flower’) or indirect approach with a lateral loop in the right atrium and a semi-deployed configuration (indirect ‘basket’) (*Figure 1A*).

Stable positioning at the medial CTI was validated anatomically in left anterior oblique 45° and right anterior 30° oblique views, aiming to obtain right atrial near-field electrograms and right ventricular far-field electrograms on the pentaspline catheter. A bolus of nitroglycerin (2 mg) was administered via the femoral venous sheath 1–5 min prior to the first PFA application. A minimum of four PFA applications were delivered, with at least two additional applications after achieving CTI block. The total number of applications was at the operators’ discretion. Twelve-lead ECG was monitored continuously, focusing on ST-segment changes. Complete bidirectional CTI block was defined according to the differential pacing technique previously described.^6^ The presence of widely spaced (>100 ms) double atrial potentials along the ablation line was also assessed.^7^

## Results

This cohort included 32 consecutive patients (*Table 1*). Cavotricuspid isthmus ablation was performed with the pentaspline PFA catheter in a direct ‘flower’ configuration for 21 patients (66%) and indirect ‘basket’ configuration for 9 patients (28%). Both configurations were used in two patients (6%). Sinus rhythm restoration was observed after the first PFA application in 17/19 patients (89%) with ongoing typical atrial flutter (*Figure 1B*). The median number of PFA applications was 6, interquartile range (IQR) 5–8 in ‘flower’ configuration, and 6, IQR 6–10 in ‘basket’ configuration. Acute bidirectional conduction block within the CTI was obtained in all patients. Widely separated (>100 ms) local double potentials alongside the ablation line could be recorded by the pentaspline catheter in 21 patients (66%) and clearly appeared after the first PFA application in 17/21 patients. Double potentials were visualized in all the nine ‘basket-only’ configuration cases and in 12/23 cases (52%) using ‘flower’ configuration, either ‘flower-only’ or ‘flower-basket’ (*P* = 0.01) (*Figure 1B*). The mean duration from insertion of the PFA catheter into the right atrium to completion and assessment of complete CTI block was 8.2 ± 2.6 min. After 20-min waiting period, none of the patients exhibited conduction recovery across the CTI. None of the patients developed atrio-ventricular (AV) conduction disturbance, ST-segment elevation, or haemodynamic instability following the PFA applications. There were no notable clinical haemodynamic consequences after nitroglycerin injection. In the five patients who underwent 3D electroanatomical mapping pre- and post-CTI ablation for illustrative purpose, the voltage maps showed extensive low voltage and propagation block on the CTI, but with normal voltage in the lateral and septal regions of the CTI, notably at the triangle of Koch (*Figure 1C*). At 6-month follow-up, 30/32 patients (94%) did not experience symptom recurrence and were in sinus rhythm on 12-lead ECG. Two patients experienced left atrial arrhythmia recurrences and underwent redo procedures with 3D electroanatomical mapping, showing persistent bidirectional block along the CTI. There was no acute coronary event during the follow-up period.

**Table 1:** Patients baseline characteristics

Baseline characteristics	All patients (*n* = 32) mean ± SD, median (IQR) or *n* (%)
Age, years	62 ± 8
Sex (male)	27 (84)
AF type	
Paroxysmal	13 (41)
Persistent	19 (59)
Indication for CTI ablation	
Documented typical AFl before the procedure	13 (41)
Typical AFl at the beginning of the procedure	8 (25)
Typical AFl induced during the procedure	11 (34)
Hypertension	17 (53)
Diabetes	5 (16)
History of stroke or TIA	2 (6)
Coronary artery disease (stent or CABG)	3 (9)
Heart failure	13 (41)
CHA_2_DS_2_-VASc	2 (1–3)
Body mass index, kg/m^2^	28 ± 4
LVEF	
LVEF > 50%	20 (63)
LVEF 40–50%	4 (13)
LVEF < 40%	8 (25)
Antiarrhythmic medications	
Class Ic	9 (28)
Amiodarone	13 (41)

AF, atrial fibrillation; AFl, atrial flutter; CABG, coronary artery bypass graft; LVEF, left ventricular ejection fraction; TIA, transient ischaemic attack.

This is the first real-life case series of consecutive patients undergoing CTI ablation using a pentaspline PFA catheter. This approach was successfully and safely performed in 32 cases. We observed a high acute efficacy with sinus rhythm restoration after the first PFA application in 89% of patients and persistence of a bidirectional CTI block after a 20-min waiting period in all. The direct ‘flower’ or indirect ‘basket’ approaches were similarly efficient with a median number of six applications. The safety profile also appeared satisfactory with no patients developing AV conduction disturbance or ST-segment elevation, under cover of a 2 mg bolus of nitroglycerin. The pentaspline PFA catheter has been developed to perform PVI only but CTI ablation is often required during AF ablation procedure. Using the same catheter to perform both PVI and CTI ablation could be time and cost-saving. Moreover, this could reduce the carbon footprint of the procedure.^8^ Some concerns have been raised about the risk of coronary spasm associated with the use of the PFA catheter in the CTI. In patients undergoing CTI ablation with a focal PFA catheter, vasospasm of the right coronary artery (RCA) was observed in four of five patients (80%) without nitroglycerin injection.^9^ Similar results were found with the pentaspline PFA catheter: subtotal vasospasm of the RCA was observed in all patients without nitroglycerin injection whereas only mild to moderate vasospasm occurred in 20% of patients after the administration of intracoronary or intravenous nitroglycerin.^10^ None of these vasospastic events resulted in ST-segment elevation or arrhythmias.^9,10^ Contrary to radiofrequency ablation,^11,12^ it appeared that PFA-induced vasospasm does not lead to any discernible degree of coronary stenosis.^13^ Intracoronary injection of nitroglycerin appeared unnecessary as an intravenous injection before the first PFA application seemed to be a safe approach.

The optimal number of PFA applications, especially after obtaining CTI block, remains to be determined in order to prevent late reconduction. The safety profile of CTI ablation using the pentaspline PFA catheter observed in our study also has to be further confirmed in larger cohorts.

## Data Availability

The data that support the findings of this study are available on request from the corresponding author, F.A.
